# Development of Hydrogen Storage Tank Systems Based on Complex Metal Hydrides

**DOI:** 10.3390/ma8095280

**Published:** 2015-09-04

**Authors:** Morten B. Ley, Mariem Meggouh, Romain Moury, Kateryna Peinecke, Michael Felderhoff

**Affiliations:** Max-Planck-Institut für Kohlenforschung, Kaiser-Wilhelm-Platz 1, 45470 Mülheim an der Ruhr, Germany; E-Mails: ley@mpi-muelheim.mpg.de (M.B.L.); meggouh@mpi-muelheim.mpg.de (M.M.); moury@mpi-muelheim.mpg.de (R.M.); peinecke@mpi-muelheim.mpg.de (K.P.)

**Keywords:** hydrogen storage, complex hydrides, modeling, thermolysis, tank design

## Abstract

This review describes recent research in the development of tank systems based on complex metal hydrides for thermolysis and hydrolysis. Commercial applications using complex metal hydrides are limited, especially for thermolysis-based systems where so far only demonstration projects have been performed. Hydrolysis-based systems find their way in space, naval, military and defense applications due to their compatibility with proton exchange membrane (PEM) fuel cells. Tank design, modeling, and development for thermolysis and hydrolysis systems as well as commercial applications of hydrolysis systems are described in more detail in this review. For thermolysis, mostly sodium aluminum hydride containing tanks were developed, and only a few examples with nitrides, ammonia borane and alane. For hydrolysis, sodium borohydride was the preferred material whereas ammonia borane found less popularity. Recycling of the sodium borohydride spent fuel remains an important part for their commercial viability.

## 1. Introduction

Over the last 15 years complex aluminum and boron hydrides have been investigated as possible hydrogen storage materials [1,2,3]. Although the composition of these materials seems to be similar, the chemical behavior is entirely different. Several complex aluminum hydrides (NaAlH_4_, KAlH_4_, Na_3_AlH_6_ *etc.*) can be decomposed at elevated temperatures and rehydrogenation is possible under technically relevant conditions using catalysts [4]. However, the decomposition temperature for complex boron hydrides (LiBH_4_, NaBH_4_) is often much higher and reversibility cannot be observed under conditions used for complex aluminum hydrides [5]. Consequently, complex aluminum hydrides can be used for technical applications where rehydrogenation of the hydrogen storage material is an important prerequisite [6]. In contrast, complex boron hydrides are favored for disposal cartridge systems, releasing hydrogen in a hydrolysis reaction at ambient temperatures [7]. These different properties result in completely different engineering as well as technical requirements. This makes the development of suitable tank systems based on aluminum or boron hydride compounds challenging.

This review describes recent research in the development of tank systems based on complex hydrides for thermolysis and hydrolysis. Thermolysis requires a heat input and care must be taken in the design of the storage tank in order to effectively distribute the heat. Hydrolysis, on the other hand, requires not only effective mixing of the complex hydride and water but also separation of the produced hydrogen gas and the slurry consisting of the decomposition product and water. While thermolysis tank systems are developed in demonstration projects, hydrolysis tank systems have already found real life applications. The review is divided into two parts, the first focuses on thermolysis, while the second concerns hydrolysis. Initially, reactor, kinetic and system models will be introduced in each part. Hereafter, specific examples with the most common complex aluminum or boron hydrides are given together with their applications.

## 2. Thermolysis

### 2.1. Tank Developments for Thermal Hydrogen Evolution

Development of a hydrogen storage tank based on complex hydrides involves different levels of research. The first level provides a screening of materials at laboratory scale. Once relevant candidates have been selected, a methodology to evaluate their performances must be established. The evaluation is based on two steps. The first one is modeling of the full-scale system and the second one is manufacturing of a prototype to experimentally evaluate the performance of the material and validate the results of the simulation. The Department of Energy (DoE) in the United States of America has given several milestones for the research on the optimal complex hydride/tank design pair (Table 1). All these technical specifications are material dependent, thus the first screening starts with a selection among available complex hydrides. Nevertheless, improvement of the scale-up system through several strategies is still possible. Among all the complex hydrides claimed for hydrogen storage, just a few of them have been chosen to develop a full-scale tank system. Since Bogdanović and Schwickardi discovered that sodium alanate can reversibly store hydrogen using a Ti-based catalyst [4], its hydrogen storage properties have been widely studied. The absorption/desorption mechanism has been found to occur through a two-step process (Equation (1)). Another system based on a mixture of magnesium nitride and lithium hydride (Mg(NH_2_)_2_-2LiH), is also reviewed. This system can reversibly store hydrogen following the mechanism described in Equation (2). The last materials studied for tank development are ammonia borane (AB, NH_3_BH_3_) and alane (AlH_3_). They are not reversible by direct hydrogenation but instead need a chemical treatment to be recovered. Nevertheless, they have attracted a lot of attention owing to their high gravimetric hydrogen capacity (19.5 wt % H_2_ for AB and 10 wt % for AlH_3_). The decomposition mechanism is described in Equations (3) and (4) for AB and AlH_3_ respectively.

(1) $${\text{NaAlH}}_{4}\overset{1}{⇌}1/3{\text{ Na}}_{\text{3}}{\text{AlH}}_{6}+\;2/3\text{ Al }+{\text{ H}}_{\text{2}}\overset{2}{⇌}\text{NaH }+\text{ Al }+\;3/2{\text{ H}}_{2}$$

(2) $${\text{2 Mg(NH}}_{2}{\text{)}}_{\text{2}}\text{+ 4 LiH}\overset{1}{⇌}{\text{LiH + LiNH}}_{\text{2}}{\text{+ Li}}_{\text{2}}{\text{Mg}}_{\text{2}}{\left(\mathrm{NH}\right)}_{\text{3}}{\text{+ 3 H}}_{\text{2}}\overset{2}{⇌}{\text{2 Li}}_{\text{2}}{\text{Mg(NH)}}_{\text{2}}{\text{+ 4 H}}_{2}$$

(3) $${\text{NH}}_{\text{3}}{\text{BH}}_{\text{3}}\to \;{\left[{\text{NH}}_{\text{2}}{\text{BH}}_{\text{2}}\right]}_{n}{\text{+ H}}_{\text{2}}\to \;{\left[\text{NHBH}\right]}_{n}{\text{+ 2H}}_{\text{2}}\to \text{ BN+}3{\text{H}}_{\text{2}}$$

(4) $${\text{AlH}}_{\text{3}}\to {\text{Al + 3/2 H}}_{\text{2}}$$

In this part, we focus on the optimization of the tank system for thermolysis by reviewing the different strategies based on the aforementioned complex hydrides selected for modeling and manufacture. Firstly, an overview is given of modeling aspects developed in order to simulate tank systems. Secondly, a focus is put on the manufacturing of scaled-up tank systems to experimentally evaluate the effects of optimization parameters and the viability of the system according to the DoE requirements.

**Table 1:** Department of Energy (DoE) requirements for hydrogen storage system for mobile application [8]. PEM: proton exchange membrane.

System Storage Parameter	Original 2010 Target	Revised 2010 Target	2017 Target	Ultimate Target
Gravimetric capacity (kg·H_2_·kg^−1^ system)	6%	4.5%	5.5%	7.5%
Volumetric capacity (g·H_2_·L^−1^ system)	45	28	40	70
Operational cycle life	1000	1000	1500	1500
Filling time (min for 5 kg)	3	4.2	3.3	2.5
Min full flow rate (g·H_2_·s^−1^/kW)	0.02	0.02	0.02	0.02
Min delivery pressure at 85 °C PEM fuel cell (atm)	8	5	4	3
Fuel purity	99.99%	99.97%	99.97%	99.97%

### 2.2. Modeling of Thermolysis Tank Systems

Lab-scale evaluation of materials for hydrogen storage differs significantly from tank development in the kg scale. In order to avoid unnecessary work in the development and construction of metal hydrides tank systems, modeling is often used as a starting process for tank evolution. Simulations are therefore performed to optimize tank design and operating parameters, but also to allow evaluation of the complete performances. For the modeling of a tank system based on complex hydrides, three sub-processes that occur during hydrogen sorption require implementation in the calculations: (i) hydrogen transport, (ii) chemical reaction and (iii) heat transfer [9]. Hydrogen transport and heat transfer are modeled through mass, momentum and energy balance equations and all simulation are performed using the same assumptions and equations. Chemical reactions are modeled using empirical kinetic models; therefore the next section focuses on the different models found in the literature. After the final model is proposed, parametric studies can be performed to gain insight into the influence of different parameters on the system performance.

The first step in building a kinetics model is determination of the dehydrogenation mechanism of the considered material. Once the mechanism is known, the rate for each step is defined by a general model parameterized using three variables: the temperature *T,* the conversion α, and the pressure *p* (Equation (5)) [10]:

*d*α/*dt* = (*A*e^−*E*a/R*T*^) × *f*(*p*, *p*_eq_) × *g*(α) (5)

where *E*_a_ is the activation energy, *A* is a pre-exponential factor, and R is the universal gas constant. The function *g*(α) and *f*(*p*, *p*_eq_) (*p*_eq_ is the equilibrium pressure) must be determined experimentally at isobaric and isothermal conditions. The choice of the function *g*(α) is determined by fitting experimental data with a chosen kinetics model. Typically, two models are used: (i) decelerating model (reaction order model) (Equation (6)) and (ii) sigmoidal model described by Johnson-Mehl-Avrami (JMA) equations, (Equation (7)).

*f*(α) = (1 − α)*^n^*; *n* = reaction order (6)

*f*(α) = *n*(1 − α)(−ln(1 − α))^(*n* − 1)/*n*^; *n* = constant (7)

Once a proper model is chosen, an assumption on the function *f*(*p*, *p*_eq_) has to be made, in order to determine both the activation energy *E*_a_ and pre-exponential factor *A*. Usually *f*(*p*, *p*_eq_) is described by the logarithm ±ln(*p*/*p*_eq_) or approximated by its Taylor development. In the literature numerous pressure dependent models have been proposed. Table 2 summarizes all the kinetic models proposed for the materials used in tank modeling.

**Table 2:** Summary of the main kinetics models with pre-exponential factor *A* and activation energy *E*_a_.

Material	Reference	Step	*g*(α)	*f*(*p*, *p*_eq_)	*A* (s^−1^)	*E*_a_ (kJ·mol^−1^)
NaAlH_4_	[11]	1_des_	1st order	ln(*p*_eq_/*p*)	1.9 × 10^11^	85.6
		2_des_	1st order	ln(*p*_eq_/*p*)	2.9 × 10^10^	88.3
		1_abs_	2nd order	ln(*p*/*p*_eq_)	6.2 × 10^8^	61.6
		2_abs_	1st order	ln(*p*/*p*_eq_)	1.0 × 10^8^	56.2
	[12]	1_abs_	JMA *n* = 1.4	ln(*p*/*p*_eq_)(*p*/*p*_0_)^2.7^	3.6 × 10^−1^	54.7
			1st order	ln(*p*/*p*_eq_)(*p*/*p*_0_)^1.5^	1.9 × 10^3^	62.9
		2_abs_	JMA *n* = 1.4	(*p − p*_eq_)/*p*_eq_	2.0 × 10^11^	117.5
			1st order	((*p − p*_eq_)/*p*)*^1.5^*	1.2 × 10^8^	86.4
	[13]	1_des_	2nd order	(*p*_eq_ − *p*)/*p*_eq_	4.0 × 10^5^	110
		2_des_	1st order	(*p*_eq_ − *p*)/*p*_eq_	6.0 × 10^12^	110
		1_abs_	2nd order	(*p − p*_eq_)/*p*_eq_	1.0 × 10^8^	80
		2_abs_	1st order	(*p − p*_eq_)/*p*_eq_	1.5 × 10^5^	70
	[14]	1_abs_	JMA *n* = 1.33	(*p − p*_eq_)/*p*_eq_	1.5 × 10^9^	91.5
		2_abs_	JMA *n* = 1.33	(*p − p*_eq_)/*p*_eq_	2.3 × 10^8^	91.7
	[15]	1_des_	0 order	((*p*_eq_ − *p*)/*p*_eq_)^2^ *+* 1.04((*p*_eq_ − *p*)/*p*_eq_)	5.4 × 10^1^°	105.8
		2_des_	1st order	((*p*_eq_ *−* *p*)/*p*_eq_)^2^ + 0.46((*p*_eq_ *−* *p*)/*p*_eq_)	3.4 × 10^8^	91.5
Li_2_Mg(NH)_2_	[16]	1_des_	JMA *n* = 1.5	ln(*p*_eq_/*p*)	2.3 × 10^12^	131.8
		2_des_	0 order	1 − (0.001515/0.33*wt*_max_)(*p* − 1.1)	3.0 × 10^15^	161.4
		1_abs_	0 order	(*p − p*_eq_)/*p*_eq_	2.7 × 10^17^	164.8
		2_abs_	1st order	(*p*_eq_ − *p*)/*p*_eq_	4.7 × 10^14^	147.8

des = desorption, abs = absorption; definition of the steps see Equations (1) and (2); *wt*_max_ is the maximum weight fraction.

### 2.3. Simulation of Thermolysis Tank Systems

#### 2.3.1. Simulations on Sodium Alanate

Na Ranong *et al.*, investigated the effects of the outer diameter in a multi tubular tank on refueling time, the heat transfer units and the total weight of the system [17]. The kinetics model from Franzen was used in the calculation [12]. Two 8 kg tank units with a reactor outer diameter of 16 mm and 60 mm were compared. Although the 16 mm reactor showed better results than the 60 mm reactor for both refueling time (290 s (16 mm) *vs.* 540 s (60 mm)) and heat transfer (610 W·m^−1^·K^−1^ (16 mm) *vs.* 319 W·m^−1^·K^−1^ (60 mm)), the 60 mm reactor was chosen for manufacture due to less number of tubes needed within the tank leading to a reduced overall weight (68 kg (16 mm) *vs.* 38 kg (60 mm)).

Hardy and Anton developed a hierarchical methodology for hydrogen loading process to refine operating parameters [18,19]. The methodology consists of four sub-models: (i) A kinetics scoping model [20]; (ii) A geometry scoping model was developed to refine length scale in a given tank geometry (the geometry is available in [21] and the calculated geometry parameters in [18]); (iii) a heat transfer removal scoping model developed to calculate coolant flowrate and temperature, pressure drop, convection heat coefficients and temperature increases along the cooling channel (the results are described in [19]); and (iv) a 2- and 3-dimensional finite element model built with previous optimized parameters (from (i) to (iii)) as input parameters. The model was used to assess the detailed performances for a NaAlH_4_ system, but could be also adapted for other systems, if a proper kinetic model would be provided.

Pfeifer *et al.*, investigated the thermal coupling between a high temperature proton exchange membrane (HT-PEM) fuel cell with a 2 kg sodium alanate tank using simulation studies in order to identify the parameters influencing efficiency and start-up during hydrogen discharge [22]. The Luo and Gross kinetic model was used in the simulation [11]. The authors highlighted that the decomposition kinetic was the limiting parameter on the efficiency of the cell. An optimum operating temperature of 185 °C with a fuel cell power of 1 kW was found. The minimum operating temperature was evaluated at 120 °C with a cumulative output of 0.8 kWh.

Bhouri *et al.*, performed optimizations of two different tank configurations: a shell, tube and fin hydride bed system (1) and a multi-tubular reactor equipped with fins (2) [23,24]. The hierarchical methodology developed by Hardy and Anton [18,19] was used with a UTRC^TM^ kinetic model [20]. The purpose for configuration (1) was to optimize the fin thickness and number of heat exchanger on both loading and discharging processes. It was demonstrated that the fin thickness had no influence during the loading process. However, during discharge the rate and amount of hydrogen release were improved by using thicker fins. After all, this decreased the gravimetric and volumetric capacities [23]. The effects of the number, thickness and tip clearance of the fins on hydrogen uptake were evaluated for configuration (2). The authors concluded that an increase in the number of fins and decrease in the tip clearance had a beneficial effect on the loading rate at the expense of hydrogen capacity. The optimized configuration (six fins, 0.1 cm thickness and 0 cm of tips clearance) led to an improvement of the loading rate by 41%. On the contrary, fin thickness had no impact on the loading rate. Thus, optimization of the system was based on a compromise between loading rate and fins contribution to the overall weight [24].

Effects of operating parameters during refueling in a cylindrical bed with longitudinal fins and cooling heat exchanger were reviewed by Raju and Kumar [25]. The geometry of the tank was adopted from Hardy and Anton [18,19]. A sensitivity analysis was performed to estimate the effect of the initial bed temperature, supply pressure, coolant flow rate, coolant temperature and thermal conductivity. The kinetic model was adopted from Luo and Gross [11]. An operating pressure of 15 MPa or above was chosen to keep the temperature in the optimal range 420–450 K and a coolant flow rate of 25 lpm/tube at 380 K was found to be optimal. The best configuration required an increase of 8 W·m^−1^·K^−1^ in the thermal conductivity by compaction with an addition of a conductivity enhancer (aluminum or graphite) [25].

Simulation was performed for a multi-tubular tank using the kinetic model from Lozano *et al.* [14]. Effect of the inner diameter, compaction and addition of expanded natural graphite (ENG) were evaluated according to hydrogen capacity during the loading (here 4.5 kg H_2_ in 10 min was used as a constraint). The optimized configuration, for a 35 mm inner diameter tank with compacted powder and no ENG addition, was determined at 10 MPa and 130 °C [26]. The effect of graphite addition and the design of the heat exchanger were also studied by Johnson *et al.* [27]. The simulation was performed using a first principle kinetic model that relied on experimentally determined parameters (full explanation of the model can be found in [20]).

In 2014, a comparative resistance analysis was developed [9], using a kinetic model from Lozano *et al.* [14,15]. The authors estimated, based on the driving force for each sub-process (hydrogen transport, intrinsic kinetics and heat transfer), the resistance during hydrogen sorption. The authors highlighted that hydrogen transport has a negligible effect (independent of the size of the reactor), the intrinsic kinetics play a decisive role in a small cell (2 mm), and heat transfer is the main resistance during absorption in a scale-up tank. Consequently, improvement of heat transfer is necessary in order to obtain a tank system viable for application.

#### 2.3.2. Simulations on Other Complex Hydrides

Ammonia borane (AB, NH_3_BH_3_) has a very high practical gravimetric density of about 16 wt % and both solid and liquid AB have been tested for application purposes [28,29]. Unfortunately, AB requires an off-board refueling system, since chemical treatment is a necessity for regeneration. The off-board regeneration system would be the limiting step in the development of AB storage tanks. A model was developed to estimate the AB efficiency in Auger and slurry reactors [28], together with a set of guidelines for the fluid-phase hydrogen storage properties [29].

The complex hydride (CH) 2LiNH_2_-1.1MgH_2_-0.1LiBH_4_-3 wt % ZrCoH_3_ and the metal hydride (MH) LaNi_4.3_Al_0.4_Mn_0.3_ were used in tandem for scale-up tank simulation studies [30,31,32]. At first, a new reactor concept was designed by combining advantages of both CH (high hydrogen content) and MH (high reaction rate) [30,31]. The authors used a cylindrical tank heated through the outer wall. The MH bed was positioned in the center of the tank separated from the CH bed by a membrane. In the absorption step, the heat of absorption of the MH bed (low temperature) was transferred to the CH bed (higher temperature). The kinetics for the simulation was based on the model by Bürger *et al.* [16]. The viability of the concept was demonstrated by comparing the temperature front in a pure CH bed and in a MH/CH combined bed. At 7 MPa and 130 °C, the absorption in the pure CH bed started from the annulus to the core. However, the heat removal (from the outer wall) was difficult when the temperature increased in the core. In the combined MH/CH bed, the MH part started to absorb at 7 MPa and room temperature. When the inner temperature exceeded 200 °C, the heat was transferred from the core to the annulus, which in turn facilitated heat removal from the outside of the tank [30]. In regard to the desorption step, the MH stabilized the pressure in the reactor around the equilibrium pressure of the CH, whereby a pressure drop was avoided during desorption [31]. In conclusion, the MH bed had a beneficial effect on the system by introducing a better temperature management during absorption and a better pressure management during desorption. In a later work, the tank concept was used in simulation studies to optimize operating parameters. The lower loading time was determined for the ratio between the thicknesses of the CH/MH beds; the optimal CH thickness was 12.5 mm. A sensitivity study demonstrated that the limiting parameter on the loading rate was the thermal conductivity of the complex hydride [32].

### 2.4. Developed Complex Metal Hydride Based Tank Systems

Na Ranong *et al.* [17] have given a comprehensive overview of design solutions for the different components of metal hydride storage tanks. A higher density of the powdered material can improve the thermal conductivity and volumetric capacity limitations [33,34]. Lozano *et al.*, found that compaction of the material is the most influential factor for the optimization of the hydrogen capacity of the storage system [26]. Some researches and developers have been concentrating their efforts on finding a suitable material and designing a system for onboard vehicular applications [27,35]. Others have focused on increasing the efficiency of the combined heat-power units for the stationary hydrogen storing systems [36,37,38], or on improving different properties and kinetics of the complete system [35,39,40]. Here, a brief overview is presented on hydrogen storage systems that have been published, based on sodium alanate, metal amides, ammonia borane and alane. The research and development efforts are aimed at improving the gravimetric/volumetric capacity, efficiency of the heat management and lowering the overall cost of the systems.

#### 2.4.1. Sodium Alanate Based Tank Systems

Bellosta von Colbe *et al.*, demonstrated the functionality of an 8 kg tank during absorption and desorption with a peak technical absorption time below 10 min, thereby validating the design and simulation work carried out previously [15,41]. The ease of use, speed of charging, and reproducibility of results of the 8 kg sodium alanate tank were highly promising [36]. This has paved the way for the application of complex hydrides based hydrogen storage tanks wherever a source of available waste heat is compatible with the energy needs of the tank (especially regarding the temperature level). This is the case especially in large scale and stationary applications [36].

Utz *et al.*, studied the behavior of a powdered bed containing NaAlH_4_ doped with 4 mol % CeCl_3_ in a lab-scale hydrogen storage tank with flow-through mode. The results showed a significant influence of the cooling by excess hydrogen on the flow-directional temperature profiles. The initial thermal conductivity of the bed increased by a factor of 1.3 compared to values reported in literature (0.67 W·m^−1^·K^−1^). This caused significantly lower temperature peaks in the center of the reaction bed. The value for the permeability decreased by 50% and led to increase in the pressure drop. No cycling degradation after 36 cycles was observed and a storage capacity of approx. 3.9 wt % H_2_ was reached with this material [39].

**Figure 1:**
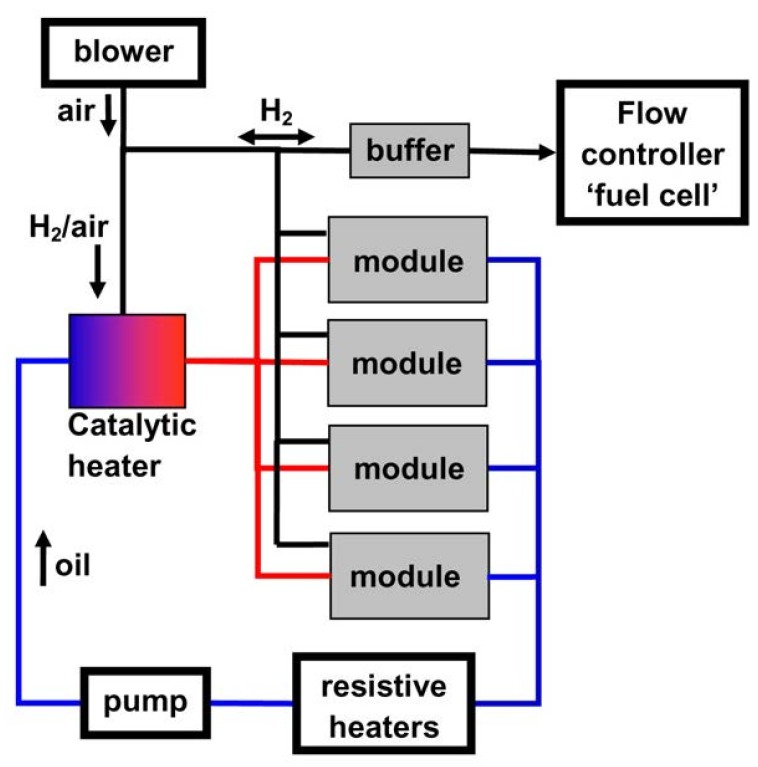
Process flow diagram of General Motors R&D/Sandia National Laboratories hydrogen storage system. Reprinted with permission from reference [27]. Copyright 2012 Elsevier.

Modularity was a key concept in the design of a General Motors R&D/Sandia National Laboratories hydrogen storage system (Figure 1). The system was designed to be refueled in approximately 10 min and to deliver hydrogen at up to 2.0 g·s^−1^. A heat amount of 60 MJ had to be removed during refueling by means of a circulating heat transfer fluid. However, for hydrogen delivery, heat must be supplied to the hydrogen storage system *in-situ* and this was achieved by means of a catalytic heater [27].

**Figure 2:**
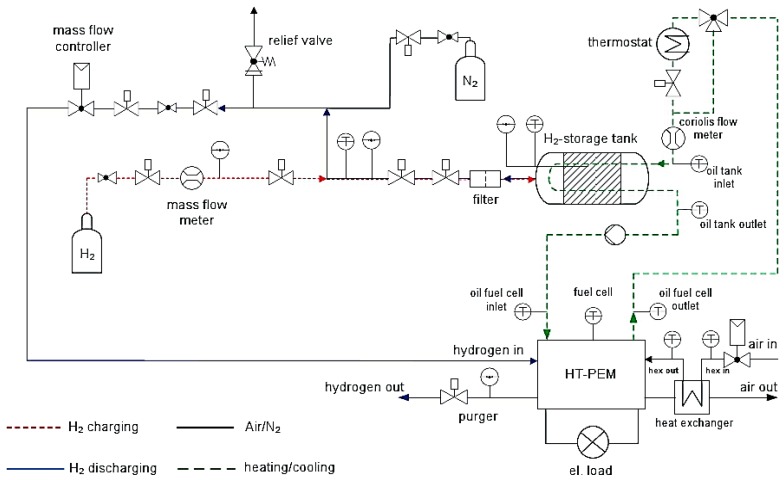
Sketch of the test rig where the storage tank is coupled with high temperature proton exchange membrane (HT-PEM) fuel cell. Reprinted with permission from reference [37]. Copyright 2011 Wiley.

Urbanczyk *et al.*, designed a hydrogen storage tank based on 2.7 kg of NaAlH_4_ doped with 4 mol % TiCl_3_ that was thermally coupled with a high temperature proton exchange membrane (HT-PEM) fuel cell (Figure 2). The waste heat of the fuel cell was used to heat up the storage tank during dehydrogenation, which was consequently fed into the fuel cell. The desorbed amount of hydrogen gas was enough for 3 h of fuel cell operation and the system produced 940 Wh of cumulative energy [37].

**Figure 3:**
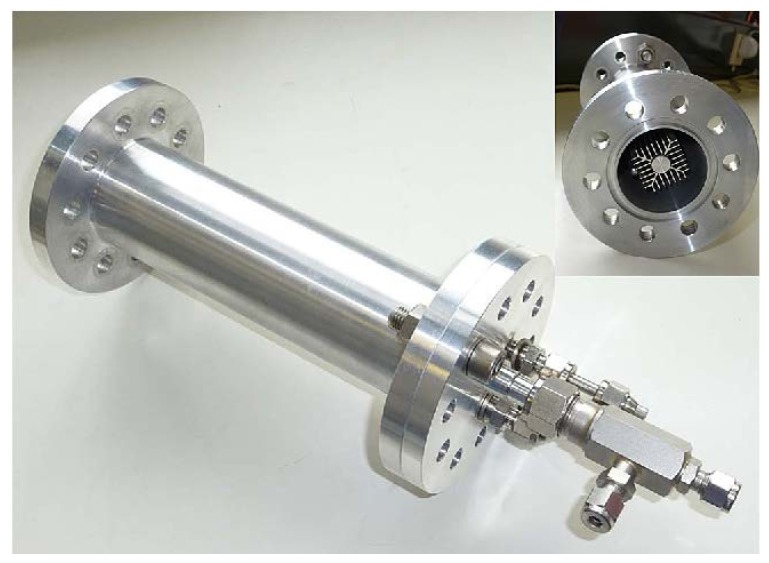
Al-Alloy hydrogen storage tank developed by Institut für Energie- und Umwelttechnik/Max-Planck-Institut für Kohlenforschung (IUTA/MPI). Reprinted with permission from reference [38]. Copyright 2014 Elsevier.

Na_3_AlH_6_ doped with 4 mol % TiCl_3_ was used as a storage material in an Al-alloy tank system developed at IUTA (Institut für Energie- und Umwelttechnik, Germany) and Max-Planck-Institut für Kohlenforschung, in order to decrease the overall weight, (Figure 3). The hexahydride was operated at a lower pressure compared to NaAlH_4_. The heat transfer was realized through an oil flow in a bayonet heat exchanger, manufactured by extrusion molding from an aluminum alloy. The 0.21 kg hydrogen storage tank released and absorbed 3.6 g (1.7 wt %) of hydrogen at approximately 450 K. A test with 45 cycles (hydrogenation and dehydrogenation) was carried out without any failure of the tank or its components. Operation of the tank under real conditions indicated the possibility for applications with stationary HT-PEM fuel cell systems [38].

The same partners at IUTA and Max-Planck-Institut für Kohlenforschung used Na_3_AlH_6_ doped with 4 mol % TiCl_3_, 8 mol % Al and 8 mol % activated carbon as a hydrogen storage material for a scaled up 1.9 kg Al-alloy storage tank with corrugated heat exchangers. Up to 31 hydrogenation and dehydrogenation cycles were performed without degradation. The aim of the demonstration project was to produce a lightweight system that could be connected to a HT-PEM fuel cell. The system could improve efficiency of a combined heat-power unit for household applications [42].

A lightweight tank for hydrogen storage containing 4.4 kg of NaAlH_4_ doped with TiCl_3_·1/3AlCl_3_ and expanded natural graphite (ENG) was designed, built and tested [36]. The tank improved the gravimetric capacity by 83% and the volumetric capacity by 49% compared to a previous tank [36]. Additionally, the heat evolution and temperature spikes during hydrogen absorption were studied and since NaAlH_4_ has a high specific heat, the material itself acted as a heat sink, aiding in the heat management of the system. The authors also performed the first-ever radiography with fast neutrons on the operational complex-hydride based test tank [43].

#### 2.4.2. Metal Amide Based Tank Systems

Yan *et al.*, demonstrated that changes in the compaction pressure and the graphite content of Mg(NH_2_)_2_-2LiH-0.07KOH pellets in a cylindrical lab-scale tank affected the hydrogen storage properties. The addition of up to 9 wt % ENG significantly enhanced the heat transfer in the hydride bed, which resulted in the rise of the central temperature. This improved the hydrogen desorption property of the tank and also increased the hydrogen desorption time at a constant flow rate (Table 3). The volumetric storage density of the composite reached 47 g·L^−1^ after compression at 365 MPa [40]. The hydrogen permeability decreased with increasing compression pressure, while the entire desorption process only changed slightly. The results indicated that the heat transfer behavior of the hydride bed dominated the hydrogen desorption of the tank [44].

Bürger *et al.*, designed a 600 g lab scale combination reactor where two materials, 2LiNH_2_-1.1MgH_2_-0.1LiBH_4_-3 wt % ZrCoH_3_ and LaNi_4.3_Al_0.4_Mn_0.3_, are separated by a gas permeable layer. As it was mentioned in the modeling part, the reason for the combined reactor concept was that the fast desorption reaction of the metal hydride stabilizes the pressure of the reactor at the equilibrium pressure of the complex metal hydride. The model was developed and validated by experimental data. This reactor has been developed in order to be coupled with a HT-PEM fuel cell running at technically relevant conditions, *i.e.*, allowing for 2 h operation of 1 kW_e_ fuel cell [31].

#### 2.4.3. Ammonia Borane and Alane Based Tank Systems

Brooks *et al.*, investigated the slurry-based ammonia borane (AB) and alane system for hydrogen storage in light-duty vehicles and developed a lab-scale slurry based reactor. AB was selected because of its promising practical hydrogen storage capacity of approximately 16 wt %, while alane has a practical hydrogen storage capacity of 10 wt % [45]. Once these chemicals have reacted to produce hydrogen, the byproduct must be removed from the system and off-board regenerated before it can be reused. This differs from the previous system described that can be regenerated directly onboard. Although slurries can be difficult to handle, they can be transported on and off the vehicle using a flow-through process [35]. There are several drawbacks in handling these materials. The high densities of both slurries result in need for mixing the feed and product prior to movement. Alane was found to have some advantages over AB, which generates fuel cell impurities (*i.e.*, ammonia, borazine, diborane) that require removal before reaching the fuel cell [46].

A summary of the developed hydrogen storage systems based on thermolysis is shown in Table 3.

**Table 3:** Summary of the developed hydrogen storage systems.

Storage Material	Weight of Storage Material (kg)	Design	Capacity (wt. % H_2_)	T, P Conditions Charging/Discharging	Kinetic/Cycles	Purpose
NaAlH_4_ doped with 2 mol % (TiCl_3_-0.3AlCl_3_), 5 mol % carbon	8	Tubular reactor with porous sintered metal tube	3.7	charging: 125 °C, 10 MPa; discharging: 160–175 °C, 0.02–1 MPa	1–10 activation cycles after 10 min 80% capacity achieved	Large scale and stationary applications [36].
NaAlH_4_ doped with 2 mol % CeCl_3_	0.087	Hydride bed reactor with flow-thru mode	3.9	charging: 130 °C, 10 MPa; discharging: 180 °C, 0.13 MPa	36 experiments; decrease in permeability; increased thermal conductivity λ_eff_ = 0.67 W·m^−1^·K^−1^	To investigate an operational principle, changes in heat transfer, permeability and reaction kinetics [39].
NaAlH_4_, Al, 10 wt % ENG	4 × 21.5	Modular system of 12 tubular vessels	3.2 in 10 min	charging: 120–150 °C, 5.52–6.89 MPa, oil temperature: 120–140 °C	40 absorption/desorption cycles	To be refueled in 10 min and to deliver H_2_ up to 2.0 g·s^−1^ [27].
NaAlH_4_ doped with 4 mol % TiCl_3_	2.7	Stainless steel tank with double wounded helical coil heat exchanger	2.24	charging: 135 °C, 10 MPa; discharging: 120–180 °C, 0.1 MPa	7 two or more hour desorption cycles coupled with FC, that supplied 165–240 W power	To couple with HT-PEM and use waste heat from HT-PEM for desorbing H_2_ from the tank [37].
Na_3_AlH_6_ doped with 4 mol % TiCl_3_	0.213	Al-alloy tank with bayonet heat exchanger	1.7	charging: 150–170 °C, 2.5 MPa; discharging: 177–180 °C, 0.65 MPa	10 absorption/desorption cycles	To develop and test lightweight Al-alloy storage tank [38].
Na_3_AlH_6_ doped with 4 mol % TiCl_3_, 8 mol % Al and 8 mol % activated carbon	1.9	Al-alloy tank with corrugated heat exchanger	2.1	charging: 160 °C, 2.5 MPa; discharging: 180 °C, 1.6 MPa	31 absorption/desorption cycles	To develop the lightweight Al-tank that is produced by extrusion molding [42].
NaAlH_4_ doped with 2 mol % (TiCl_3_-0.3AlCl_3_) and 5 mol % ENG	4.4	Ti-alloy tube-and-shell tank system	4	charging: 124 °C, 10 MPa; discharging: 120–170 °C, 9 MPa (constant flow)	33 cycles, 120 min of absorption with restricted H_2_ flow of 245 L_n_·min^−1^; 200 min desorption 3.7 L·min^−1^	To improve gravimetric and volumetric capacity [43].
Mg(NH_2_)_2_-2LiH-0.07KOH with 9 wt % ENG	0.098	Cylindrical lab-scale hydrogen storage tank with porous sintered metal tube as H_2_ supply	N/A	charging: 220 °C, 8 MPa; discharging: 220 °C at constant H_2_ flow rate of 0.6 L·min^−1^	Desorption duration of 79.5 min at 0.6 L·min^−1^ H_2_	To investigate influence of graphite content and compaction pressure on desorption properties [44].
LiNH_2_-MgH_2_-LiBH_4_ 3 wt % ZrCoH_3_ (in annulus) LaNi_4.3_Al_0.4_Mn_0.3_ (in core)	0.6	Tubular reactor, two materials separated by a gas permeable layer	N/A	charging: 165–170 °C up to 0.17 MPa; discharging at constant and periodic H_2_ mass flow	10% of H_2_ desorbed in about 30 min; majority of H_2_ desorbed in about 1 h	To validate a model and study effects of the reactor concept on desorption performance [31].

## 3. Hydrolysis

Hydrogen release can also occur apart from being subjected to heat via hydrolysis by reaction with water. A considerable amount of work has been invested in experimental testing of hydrolysis systems for complex metal hydrides [47]. Sodium borohydride, NaBH_4_, is one of the most tested materials for hydrolysis, (Equation (8)) [48,49,50]. NaBH_4_ was first discovered in 1940 by Schlesinger and Brown, but the work was classified and not published until 1953 [51]. NaBH_4_ has a hydrogen content of 10.8 wt % and is the only complex hydride that so far has a viable field of applications.

NaBH_4_ + 2 H_2_O → NaBO_2_ + 4 H_2_ (8)

Ammonia borane (NH_3_BH_3_, AB) has received considerable interest as a hydrogen storage material owing to its high hydrogen content (19.5 wt %) and the ability to release hydrogen at low temperatures. When in contact with water, ammonia borane releases hydrogen according to Equation (9), which is accelerated in acidic conditions and by the use of metal catalysts [52,53,54,55,56]. Some work has been reported for hydrolysis of other metal hydrides or complex metal hydrides, e.g., LiH, MgH_2_, CaH_2_, LiBH_4_, KBH_4_ and LiAlH_4_ [57,58,59,60,61,62,63].

BH_3_NH_3_ + 2 H_2_O → NH_4_BO_2_ + 3 H_2_ (9)

### 3.1. Tank Developments for Hydrolysis Based Reactors

Experimental hydrolysis systems are based on two different reactor designs, a batch or flow reactor. In a batch reactor, the complex boron hydride and the catalyst are mixed together in water and the reactor is refueled by exchanging the entire solution including remaining boron hydride, byproduct and the catalyst [64,65,66,67]. In a flow reactor, the boron hydride solution is passed over or through a solid catalyst bed followed by separation of the produced hydrogen and the byproduct solution [48,68,69]. In both reactor systems, the concentration of the boron hydride solution as well as handling of the waste solution poses problems, e.g., for NaBH_4_ and NaBO_2,_ since the solubility of NaBH_4_ in alkaline water solutions is much higher than for NaBO_2_ [50,70]. The hydrolysis reaction can also occur by a reaction between steam and a solid boron hydride [57,71,72]. The use of steam can possibly reduce the amount of water otherwise used in the boron hydride solutions, thus improving the total energy storage of the system [57].

#### 3.1.1. Flow Reactors

In 2000, a first example of a flow catalyzed hydrolysis reaction was published [48]. The hydrolysis reaction was carried out over resin beads coated with Ru metal. In another study, a conversion close to 100% was achieved with a small reactor filled with an alumina support covered with Ru metal. However, the catalyst was present in excess, which may have enabled the high conversion [73]. High conversion of 95% over 700 h was reported for an auto-thermal fixed-bed reactor using a Ru-based catalyst from Millennium Cell Inc. (Eatontown, NJ, USA) [74]. The reactor used the heat evolved from the exothermic reaction between NaBH_4_ and water to sustain the hydrolysis reaction [69]. This system was further developed by integrating a heat exchanger to heat up the fuel feed prior to contact with the catalyst bed. The integrated reactor equipped with a heat exchanger gave a more uniform heat distribution over a range of fuel flow rates, which increased the catalyst effectiveness [68].

Recently, a new concept reactor with a magnetic containment of permanent trade magnets to recollect the catalyst made out of a magnetic support coated with Ru particles was described [75]. The reactor should allow better control of the hydrogen production kinetics. The reactor had an efficiency up to 90% and the catalyst showed an increased stability and activity for hydrogen generation [76]. The magnetic reactor was also tested in combination with a fuel cell [77].

The flow reactors discussed so far have a tubular design. Recently, a new π-shaped reactor was proposed [78]. A gas channel was situated above the cavity for the catalytic hydrolysis reaction. The design of the reactor decreased the impact of the hydrogen on the surface of the catalyst, improving the conversion efficiency and allowing for a stable hydrogen generation rate. The hydrogen gas was collected in the gas channel and was quickly moved away from the catalyst. This stopped the reverse flow of the NaBH_4_ solution and a continuous flow to the catalyst was achieved. The π-reactor improved the conversion with approximately 10% compared to a conventional tubular reactor [78].

Micro-reactors have also been proposed for production of hydrogen from hydrolysis of chemical hydrides for portable customer electronics [79,80,81,82]. The micro reactor described by Kim *et al.*, was made from three layers of photosensitive glass [79]. The catalyst support made of nickel foam was positioned between the glass plates. After assembly of the micro reactor, a Co-P-B catalyst was deposited on the catalyst support by reduction of a solution of 0.4 M CoCl_2_ and 0.4 M NaH_2_PO_2_ by NaBH_4_. The fuel solution was a mixture between 15 wt % NaBH_4_ and 5 wt % NaOH and the fuel feeding rate was 30 μL·min^−1^. The micro reactor had an average conversion efficiency of 93%. The micro reactor was used in combination with a micro fuel cell. The system had a maximum power output of 157 mW at a current of 0.5 A. There was no difference in performance when hydrogen supplied from the micro reactor was exchanged with pure hydrogen, which showed the micro reactor generated enough hydrogen for the fuel cell [79,80].

Zhu *et al.* introduced a micro reactor system for generating hydrogen from a NH_3_BH_3_ solution. The micro reactor employed self-circulation by a micro pumping mechanism, which was made possible by the hydrogen bubbles produced in the hydrolysis reaction [82]. This meant hydrogen was generated without parasitic power consumption. In the system, the dissolved NH_3_BH_3_ was feed into the micro reactor from a rechargeable fuel reservoir. The system was made from 375 μm thick silicon wafers using a microfabrication process and the channels were drawn by a reactive ion etching using SF_6_ and finally etched with a KOH solution. Platinum black was electroplated into the channels to serve as catalyst for the hydrolysis of NH_3_BH_3_. A dilute solution of 1 or 5 wt % NH_3_BH_3_ was used for hydrogen production and conversions up to ~52% and 57%, respectively, were recorded. A small PEM fuel cell was also used in combination with the micro reactor and allowed the generation of power for micro-scale energy devices [82].

#### 3.1.2. Batch Reactors

Ferreira *et al.*, tested two different batch reactors with a difference in volume and in the shape of the bottom of the reactor. The first reactor had a flat bottom and *V* = 0.646 L, while second reactor had a conical bottom geometry and *V* = 0.229 L [64]. Their tests were carried out with the same amount of catalyst in each reactor and the results indicated that a higher pressure in the smallest reactor (*p*_small_ = 1.14 MPa, *p*_big_ = 0.4 MPa) promoted the hydrolysis reaction. The conical shape of the smallest reactor was thought to increase the hydrogen production rate ($r_{{\text{H}}_{2,\text{ small}}}$ ≈ 0.00077 MPa·s^−1^, $r_{{\text{H}}_{2,\text{ big}}}$ ≈ 0.00036 MPa·s^−1^) [64]. In a later publication, Ferreira *et al.*, tested three different reactors, a flat bottom reactor with *V* = 0.646 L, a flat bottom reactor with *V* = 0.229 L and a conical shape reactor with *V* = 0.229 L. The conical shaped reactor was shown to be superior and increased both the hydrogen yield and generation time while decreasing the induction time. The better performance of the conical shaped reactor may be attributed to an enhanced contact between the NaBH_4_ and the catalyst [70].

#### 3.1.3. Reactor Modeling of Hydrolysis Tank Systems

Only limited reports have been published of reactor modeling for hydrolysis purposes. The physical processes inside a hydrolysis reactor are complex and multi-phase flow, heat and mass transfer need to be evaluated. Additionally, the reaction kinetics has to be considered, making the theoretical prediction/description of a reactor a complex task.

**Figure 4:**
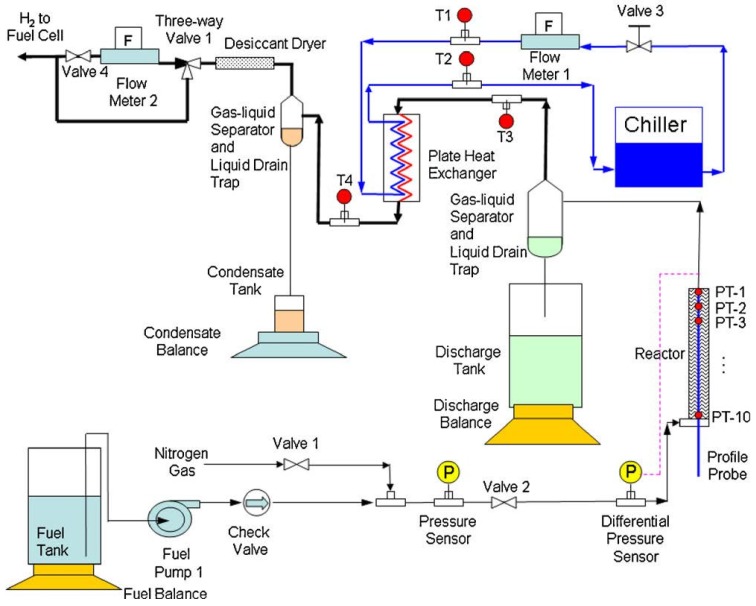
Schematic of the 1 kW_e_ NaBH_4_ hydrogen generation system. Reprinted with permission from reference [83]. Copyright 2007 Elsevier.

Zhang *et al.*, designed, built and tested a 1 kW_e_ NaBH_4_ hydrogen generation system (Figure 4) [83,84]. Experimental data were first collected and evaluated for a stainless steel packed-bed reactor with a commercial catalyst, 3% 2 mm Ru on carbon extrude and using solutions of NaBH_4_ with different concentrations. The 15 % concentrated NaBH_4_ solution gave a 95% chemical conversion with a maximum gravimetric capacity of 3.1 wt % H_2_. However, above a NaBH_4_ concentration of 15% the discharge product (NaBO_2_) would crystallize upon cooling to room temperature making it difficult to be removed from the discharge tank. The ideal NaBH_4_ concentration was suggested to be between 10% and 15%. Thermal runaway was encountered but could be avoided by continuously cooling the system. The tests also showed that different flow rates had an impact on the temperature profile inside the reactor [83].

The results from Zhang *et al.*, were further evaluated and used in a one-dimensional numerical reactor model [84]. The model assumed homogeneous catalysis and adiabatic operational conditions. The data used for the geometrical evaluation of the model was the temperature inside the reactor at different positions. The model produced outputs in the form of temperature, chemical conversion, relative humidity and molar flows of hydrogen and water vapor, which all matched the experimental data well at different NaBH_4_ concentrations. Additionally, the model included two sub-models, which allowed the evaluation of non-isothermal water evaporation processes and the pressure drop of the two-phase flow through the porous catalyst. The model was one-dimensional and therefore the geometrical parameters of the reactor were not evaluated in detail and only in the flow direction. The system was initially built for vehicle applications, but it could have potential for other small portable applications [84].

In 2012, Sousa *et al.*, published theoretical work on a 3-dimensional batch reactor model [85]. Experimental data were collected from a batch reactor at different temperatures with a very dilute alkaline solution of NaBH_4_ in order to avoid problems with solubility of the hydrolysis reaction product. The data were fitted using three different kinetic models in order to evaluate the kinetics of the hydrolysis reaction. The catalyst used in the reactor was a Ru catalyst supported on Ni-foam. At low temperatures or high NaBH_4_ concentrations the kinetics followed a zero-order model, while at high temperatures or low NaBH_4_ concentrations, the reaction depended on the NaBH_4_ concentration and the kinetics were best described by a first order model. The experimental data were further used in a numerical 3-dimensional non-isothermal model for a pilot scale reactor. This model described the transport phenomena and also included the kinetic model for the hydrolysis reaction. Mechanical stirring in the batch reactor was shown to have an impact on the mass transfer, since NaBH_4_ was more easily allowed to reach the catalytic sites. Furthermore, the model showed that NaBH_4_ in the bottom of the reactor was used less efficiently, because the produced hydrogen decreased the stirring in the bottom. The model assumed a single-phase flow during the hydrolysis reaction, which neglected the flow of hydrogen from the catalyst after production. The gas bubbles of hydrogen were responsible for the movement of the fluid and without the incorporated artificial mixing the performance predicted by the model would be extremely low and not compatible with the experimental data obtained with the reactor. In conclusion, the 3-dimensional model was able to simulate the reactor processes and validate the kinetic model [85].

In order to evaluate a two-phase flow inside a batch reactor and better describe the impact of hydrogen formation, a new two-dimensional reactor model was recently described [86]. Data obtained from a previous described experimental setup was reused for the new model [85]. The catalyst was again a Ru catalyst supported on Ni-foam. The previous model only focused on the area, where the reaction took place and the hydrogen storage region was not considered, whereas in the new model this is taken into account [85]. The new model used a two-dimensional plane through three reaction tubes, which enabled the analysis of three regions namely the reactive solution, thermal fluid and the metal walls. Therefore, the model allowed for better description of the impact of hydrogen production (bubbles) from the hydrolysis reaction. The computational methods indicated that even though a porous support (Ni-foam) was used for the catalyst, the hydrolysis reaction occurred mainly on the surface of the catalyst foam. Additionally, the results showed that only 30% of the catalyst was effectively used. Different positions of the catalyst foam as well as a plastic support for the Ni-foam inside the reactor were also studied in order to obtain information about the optimal position for reaching the quickest conversion. The optimal position was in the central region of the reactor chamber. Interestingly, the plastic support seemed to have a positive effect on the mixing during hydrogen production. The best result was obtained for a plastic support with multiple small holes. The authors concluded that a two-phase flow model approach gave a better representation of the real system compared to a single-phase flow model [86].

As mentioned earlier the hydrolysis reaction can also be carried out using steam and solid state NaBH_4_ with an added catalyst. In one report, the reactor for this type of reaction consisted of brass mesh to contain the solid NaBH_4_ inside a cobber reactor. The steam was then passed into the reactor, where the hydrolysis reaction occurred [57]. A two-part dissolution-reaction model was proposed for a steam hydrolysis system [72]. The model accounted for deliquescence, solid dissolution and hydrolysis in order to release hydrogen and the model could give reasonable estimates for the kinetic constant and the mass transfer coefficients. However, the model could as such not be used for reactor design.

#### 3.1.4. System Modeling for Hydrolysis Setups

The kinetic model described by Sousa *et al.*, was further used in a model for a PEM fuel cell power system using a NaBH_4_ batch reactor as hydrogen generator [85,87]. However, the actual model of the reactor was described with molar balance equations (zero dimension model) for the number of moles of NaBH_4_, H_2_O and NaBO_2_. This was possible because the only variable in the reactor was the hydrogen pressure. The Langmuir-Hinshelwood model was selected for describing the kinetics of the hydrogen generation rate over a range of temperatures and in time [85]. The results showed the possibility of using a NaBH_4_ hydrolysis reaction as the hydrogen source for the PEM fuel cell system. Indeed, this study had a bigger perspective, as a complete NaBH_4_-PEM fuel cell power system was simulated. The magnetically supported reactor described by Pozio *et al.*, was also simulated with a simple mathematically model. This was used to evaluate the process of catalytic hydrolysis and in a later publication the complete hydrogen generator was simulated [75,76].

### 3.2. Applications Using Sodium Borohydride

NaBH_4_ has been given a no-go recommendation as a solid-state hydrogen storage material for automotive applications by the US DoE due to its high decomposition temperature. However, since NaBH_4_ releases hydrogen by hydrolysis at room temperature and the solution is both stable and non-toxic, NaBH_4_ has a large potential for defense and civil applications. For application purposes, hydrogen is usually catalytically generated from alkaline NaBH_4_ solutions. An advantage of using NaBH_4_ is that no external heat supply is required due to the exothermic hydrolysis reaction. A disadvantage is that the NaBO_2_ byproduct (although environmentally friendly), needs to be collected in a separate tank in order to be recycled off board into NaBH_4_. For more information on the role of NaBH_4_ in the field of hydrogen fueled applications, the reader is referred to more in-depth articles and reviews [7,88,89,90,91].

#### 3.2.1. Small-Unmanned Aerial Vehicles

Small-unmanned aerial vehicles (UAVs) have gained much interest in the fields of defense and security in order to minimize human loses. They have been used for surveillance missions owing to the low noise and heat emissions, minimizing detection by enemies. Instead of internal combustion engines or secondary batteries, which have low thermal efficiencies and face short flight endurance (60–90 min), fuel cell systems are regarded as a more suitable alternative power source. Fuel cell systems show high thermal efficiencies, have an electrochemical reaction rather than a combustion reaction, and silent mode of operation [92].

As fuel cells operate on hydrogen, compressed hydrogen [93], liquefied hydrogen [94,95,96,97], and chemical hydrides [94] have all been used for UAV applications. For example, AeroVironment Inc. (Monrovia, CA, USA) was the first to use liquefied hydrogen for a high-altitude long-endurance (HALE) aircraft vehicle (Global Observer), which operated for over an hour. However, the manufacturers claimed 24 h flight time with a full tank [94]. In the case of compressed hydrogen, Bradley *et al.*, reported 43 min of cruising flight, based on the measured capacity of the on-board hydrogen tank (192 L), which could be increased to 52 min, if the hydrogen utilization was increased to 99% [93]. Since chemical hydrides have high energy densities, the combination with a proton exchange membrane (PEM) fuel cell stack can significantly improve the flight duration of UAVs compared to liquefied or compressed hydrogen [98]. In this section various small UAVs, which use NaBH_4_ as their hydrogen source, are discussed as to their design, manufacture, and flight tests.

The US Air Force Research Laboratory in conjunction with Small Business Innovation Research partners AeroVironment, Protonex Technology Corporation (Southborough, MA, USA) and Millennium Cell Inc. successfully demonstrated a nearly 5 h flight with Puma, an UAV, operating on a lithium ion battery and a PEM fuel cell system using NaBH_4_ as a hydrogen source [94]. The flight duration was increased to 9 h through further technological developments, demonstrating the improvement in endurance using chemical hydrides instead of compressed hydrogen [99]. The lithium ion battery provided the power for take-off and maneuvers whereas the fuel cell recharged the battery and provided a continuous power supply for the plane during cruise flight. Weighing only 5.7 kg and with an aerial observation at line-of-sight up to 10 km, the Puma was ideal for military applications [99].

**Figure 5:**
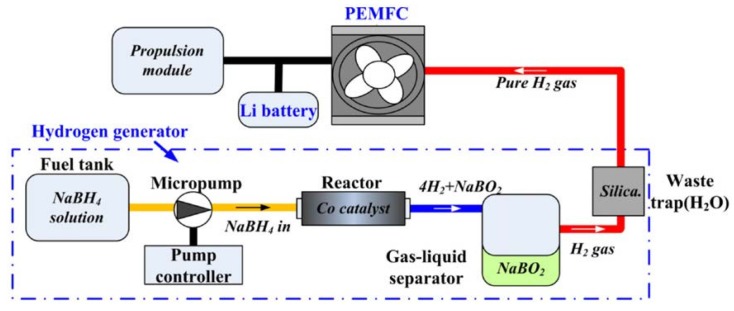
Operating principle of fuel cell system equipped in the unmanned aerial vehicle (UAV) platform. Reprinted with permission from reference [98]. Copyright 2011 Elsevier.

Kim *et al.*, also reported the use of a PEM fuel cell system operating with NaBH_4_ for UAVs, which consisted of a fuel cell stack, hydrogen generator and a hybrid power management system (Figure 5) [98]. Catalytic hydrolysis resulted in the formation of hydrogen gas and borate by feeding the 15 wt % NaBH_4_ alkaline solution through the catalytic reactor. The generated hydrogen was purified by a dehumidifier and used to power up the fuel cell stack and Li battery, whereas NaBO_2_ was separated in a gas-liquid separator where it remained for collection [98].

**Figure 6:**
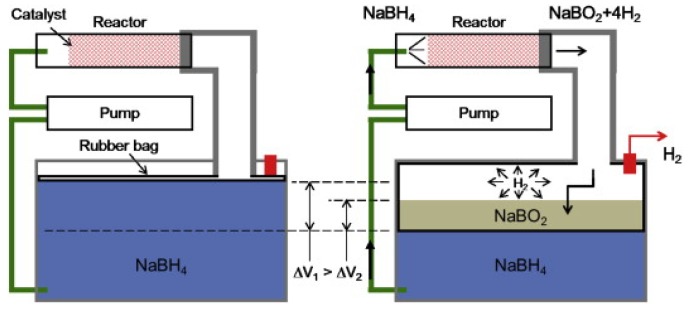
Schematic of a volume-exchange fuel tank. Reprinted with permission from reference [100]. Copyright 2014 Elsevier.

To ensure a stable performance of the fuel cell system, cooling of the stacks becomes necessary. Kim *et al.*, recommended air-cooling systems opposed to water-cooling systems to reduce complexity and overall bulkiness of the cooling system. Air flowed through the intakes, cooled the stacks and then hot air left through the exits located near the propeller. Additionally, both ground and flight tests were performed, showing a flight endurance of 2.5 h with 360 g of a 15 wt % NaBH_4_ solution [98]. A flight endurance of 2 h was achieved using 300 g of a 15 wt % NaBH_4_ aqueous solution, although, the authors claimed a flight endurance of more than 3 h would be achievable based on the energy density of the fuel cell system. However, various problems were encountered during the flight tests, which occurred in the pump, reactor and filters [92]. The design was later improved by replacing the two tank system for a volume-exchange fuel tank where the fuel (NaBH_4_ solution) and spent fuel (NaBO_2_) exchange volume within one tank hence minimizing the volumetric density (Figure 6) [100].

In order to obtain a high gravimetric energy density, the fuel cell stack and hydrogen generator should be ideally reduced to a minimum. However, the weight of the fuel cell stack typically takes up between 43% and 49.6% of the total weight in the fuel cell of the UAV [92,98,100]. Developing new materials to reduce the weight of the graphite bipolar plates incorporated in the fuel cell stacks would increase the flight endurance significantly [92].

#### 3.2.2. Small Portable Applications

Fuel cells as an alternative for lithium-ion batteries for laptops and mobile phone applications have attracted considerable research interest. This is mainly due to the low capacity of the current lithium-ion batteries, resulting in short operating times. Fuel cell technologies are regarded as the most suitable for small portable applications. Although direct methanol fuel cells are typically used, issues regarding fuel crossover and low system volumetric densities remain to be resolved [101]. The direct hydrogen fuel cell systems that use a metal hydride based hydrogen storage tank have gained much attention owing to the high power and volumetric energy densities that could be obtained [102,103]. Unfortunately, their size requirements were too large to surpass current Li-ion secondary batteries and therefore miniaturization of the fuel cells is important to achieve commercial implementation [104].

**Figure 7:**
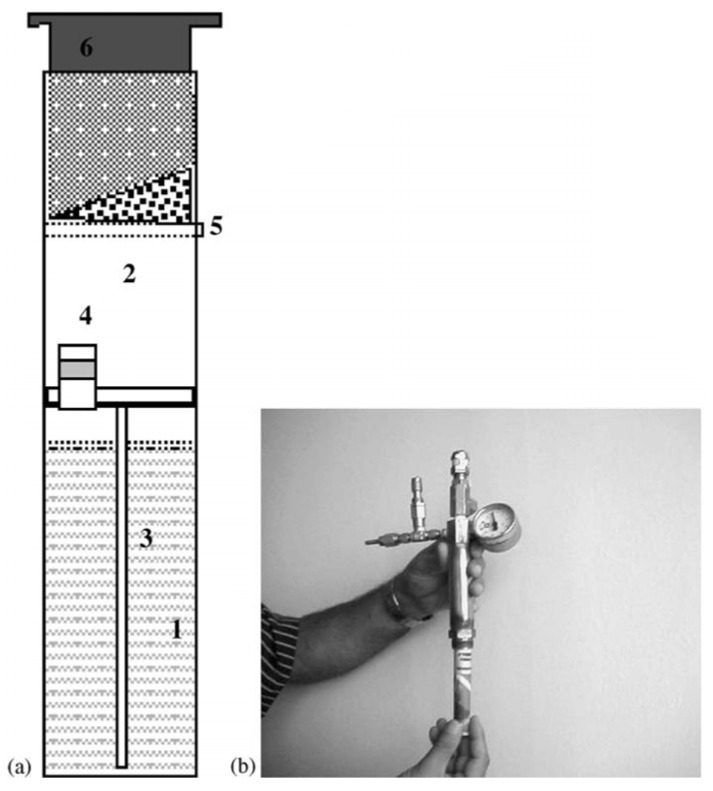
(**a**) A schematic of the hydrogen producing device: 1, HCl chamber; 2, reaction chamber; 3, connection channel; 4, pressure regulation valve; 5, diaphragm; 6, activation button; and (**b**) insertion of the device in the reaction chamber. Reprinted with permission from reference [105]. Copyright 2006 Elsevier.

NaBH_4_-based refuel devices for laptops and mobile phones have also been reported [105]. For example, Prosini *et al.*, designed a refill device to generate hydrogen from HCl/H_2_O/NaBH_4_ solution for a fuel cell powered mobile phone (Figure 7). With a 0.8 V fuel cell, a 1 W hydrogen mass flow was released for over 2.5 h. The refill case was made out of plastic and therefore the refill costs were based on the price of NaBH_4_ (1.5 g for 9–10 days). The authors claimed an estimated energy density of roughly 720 Wh·kg^−1^ instead of 170 Wh·kg^−1^ typically for commercial lithium-ion batteries, making the device competitive with existing batteries [105]. As far as we are aware, no other publications on sodium borohydride based refuel devices for small cellular or laptop applications have been published.

#### 3.2.3. Submarine Applications

**Figure 8:**
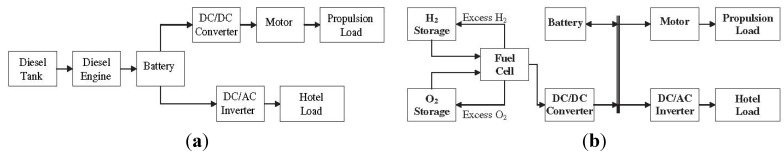
Schematic diagram for the (**a**) conventional battery based system and **(b**) fuel cell/battery-based system. Reprinted with permission from reference [107]. Copyright 2011 Elsevier.

Submarines are valuable assets for naval applications owing to their invisibility when submerged under water [106,107]. Unfortunately, most of the submarines are fitted with diesel-electric propulsion resulting in limited submerged time due to the limited capacity of the batteries. In addition, when charging the batteries only a few meters below the surface (snorkeling) the submarine is susceptible to detection. Therefore, the development of air independent propulsion in order to prolong the underwater performance becomes crucial [107,108]. Among the various investigated systems, low temperature PEM fuel cells were shown to be the most promising. A schematic diagram for a conventional battery-based system and fuel cell/battery-based system is shown in Figure 8. NaBH_4_ allows a higher gravimetric hydrogen storage capacity compared to compressed hydrogen and does not have the safety and reliability constrains of compressed gases [108,109]. Liquid oxygen can be used as the oxidant, although compressed air, which is often used for other purposes on-board, is also considered [108]. System modeling for different fuel cell/battery-based systems showed that the total submerged endurance was substantially enhanced using fuel cells [108]. Furthermore, with the NaBH_4_/liquid oxygen system the submarine could remain submerged minimally for 6.5 days with a total distance of 3500 km compared to an only 20 h submerged period for the conventional system [107].

Apart for space applications, direct borohydride fuel cells (DBFCs) in combination with H_2_O_2_ are also investigated as high-density power sources for autonomous underwater vehicles [110,111,112]. In liquid form, H_2_O_2_ is a thousand times denser than oxygen [113]. The US Office of Naval Research (Arlington, VA, USA) and the UK Defence Science and Technology Laboratory (Salisbury, UK) designed a fuel cell operating on the NaBH_4_/H_2_O_2_ system [110]. Protonex is also currently developing an underwater fuel cell power system using the Millennium Cell Inc. hydrogen generation system [114].

### 3.3. Recycling of NaBH_4_ Spent Fuel

NaBH_4_ is non-reversible and recycling of the byproduct sodium metaborate (NaBO_2_) becomes critical. A cheap and efficient route for recycling the spent fuel (NaBO_2_ dissolved in alkaline aqueous solution) still has to be developed. For NaBH_4_ decomposed in water solution, its regeneration starts with dry NaBO_2_. Therefore, the dissolved NaBO_2_ has to be dried, which is an energy intense process. NaBH_4_ can be recovered from NaBO_2_ through ball milling using MgH_2_/Mg. Here, the small particle size and high surface area increase the yield of the reaction [53,112,115].

For the direct borohydride/peroxide fuel cell, Miley *et al.*, developed a regenerative fuel cell, which can transform the end products back into the reactants, using an aqueous NaBH_4_ solution and H_2_O_2_ [113]. Prevention of the generation of hydrogen and oxygen gas during regeneration is crucial as the regeneration of NaBH_4_ and H_2_O_2_ (Equation (10)), which occurs at 2.23 V, is in competition with the electrolysis reaction (Equation (11)) that occurs at 1.23 V.

NaBO_2_ + 6 H_2_O → NaBH_4_ + 4 H_2_O_2_ (10)

2 H_2_O → 2 H_2_ + O_2_ (11)

The authors circumvented this issue by using high over-potential materials for the electrodes and exploring the formation of perborate that hydrolyze to peroxide at 1.7 V (Equation (12)), greatly reducing the gas evolution.

NaBO_3_ + H_2_O → NaBO_2_ + H_2_O_2_ (12)

Furthermore, the inhibition of the NaBH_4_ crossover, a critical issue often faced for DBFCs, can be reduced by improving the membrane electrode assembly or the liquid diffusion layer [112]. In order to further develop NaBH_4_ hydrolysis systems for portable applications, additional research is needed to reduce the cell volume, increase the gravimetric hydrogen storage capacity and reach efficient recycling of the spent fuel.

### 3.4. Applications Using Ammonia Borane

**Figure 9:**
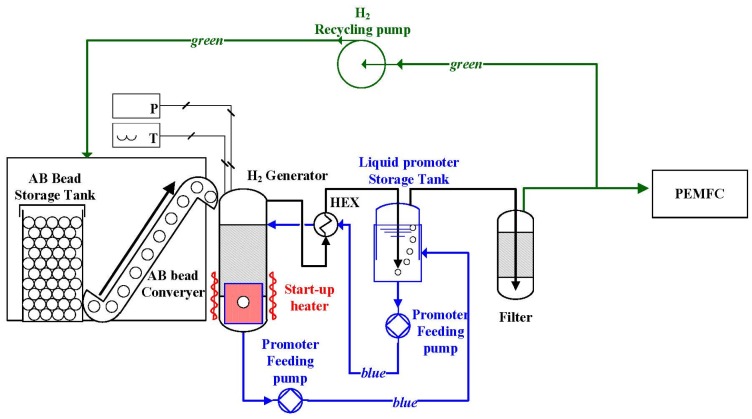
Schematic of the advanced fuel cell system powered by ammonia borane for prolonged operation. Reprinted with permission from reference [116]. Copyright 2014 Elsevier.

The development of a continuous hydrogen generator, using a continuous feeding of ammonia borane as both fuel and spent fuel, has been the focus of many researchers [28,116,117,118]. Unfortunately, high costs and raw material scarcity only allow ammonia borane to be used only for small-scale applications. Seo *et al.*, were the first to report on the design and testing of an ammonia borane hydrogen power-pack, which operated an UAV for 57 min using a 200 W PEM fuel cell. The system was operated by delivering spherical solid ammonia borane beads into a semi-batch type reactor filled with tetraethylene glycol dimethyl ether, a liquid promoter. Hydrogen purification equipment with a high filter capacity and efficient drainage system for spent fuel were also integrated for application purposes. The hydrogen generation system was the heaviest part of the hydrogen power pack taking up 64% of the total weight, whereas the fuel cell stack took up only 17.6% and the ammonia borane beads 4%. Based on the obtained results, the authors proposed an advanced reactor concept (Figure 9). They also suggested optimization for the discharging of liquid/solid spent fuels by using triethylene glycol dimethyl ether as the liquid promoter as it could enhance the fluidity of the spent fuels [116].

## 4. Summary and Outlook

There are several limitations for metal hydride-based onboard storage systems that prevent their implementation: low gravimetric and volumetric capacities, insufficient kinetics within appropriate temperatures and pressure ranges, and high cost of the overall engineering system. Modeling provides a powerful tool for the development of strategies and improvement of full-scale tanks. Several studies on tank modeling for thermolysis have been proposed, mainly based on sodium alanate with few examples of others complex hydrides. The main purpose of these simulations was the optimization of some operating parameters (temperature, pressure, thermal conductivity, coolant flow rate and coolant temperature) as well as the tank design (length scale, geometry and fins content). Optimizations were then proposed on the basis of the simulation results. Several modeling tools have been built on the basis of the hierarchical methodology and resistance analysis in order to estimate performances of the system and the limiting factors. The simulations showed that a good thermal management was necessary for the absorption, whereas the pressure control was important for desorption. Still, differences exist between the simulations, which come from the choice of the kinetic model that is implemented as the governing equation for the simulations. Therefore, precautions have to be taken, since the kinetic model is built through experimental considerations. Heat transfer in the metal hydride tank can be improved using heat exchangers, multi-tubular tank geometries and heat transfer enhancers. However, this will also increase the weight of the system. Hence, optimizations are often a compromise between heat transfer and hydrogen content. The strategy based on CH/MH beds could provide a new step in tank design leading to better temperature and pressure management without decreasing the hydrogen capacity excessively.

Although ample research has been performed with complex metal hydride materials on small-scale laboratory batches, considerably fewer full-scale thermolysis tanks/systems have been developed and analyzed. Improving the ratio between the mass of the complex metal hydride bed to the mass of the tank wall, by screening lighter materials for the tank wall and developing hydrogen storage materials exhibiting both higher gravimetric and volumetric storage properties, should be a goal in order to obtain lightweight storage systems. There are several design principles that must be taken into account while developing a complex metal hydride storage tank, such as packing arrangement of the material, hydrogen supply, heat transfer to the heat transfer medium, effective heat conductivity of the metal hydride bed and volume expansion. Additionally, efficient removal of the reaction heat from the metal hydride tank during refueling and how to provide heat during hydrogen delivery to the fuel cell remain an unsolved problem. Thermolysis of complex metal hydrides has yet to demonstrate the fulfillment of the DoE requirements (Table 1). Especially concerning the gravimetric capacity of approximately 1.5 wt % and refueling time of 10 min.

In hydrolysis, recycling of the spent fuel remains the single biggest challenge. For NaBH_4_, it requires the recycling of NaBO_2_ back into NaBH_4_, either starting from dry NaBO_2_ or through the use of a regenerative fuel cell. This often means additional costs cannot be avoided. Hence, the use of NaBH_4_ for portable applications becomes less attractive. As the byproduct from the hydrolysis reaction can decrease the catalyst efficiency, flow reactors holds the biggest potential for reaching commercial applications as these allow to collect the byproduct in a separate tank [98,100] The flow reactor with a π-shape design contained a gas channel for more efficient collection of the hydrogen gas. This design may also improve larger scale systems without sacrificing the overall volume and mass of the system. A batch reactor for hydrolysis is simple and does not allow for major design changes. Indeed, batch reactors are probably best suited for convenient tests of new catalysts and for small systems, where volume constraints do not allow for a second tank for collection of the byproduct. Implementation of a conical bottom in the batch reactor was shown to have a positive effect on the hydrolysis reaction. Tank modeling for hydrolysis systems has only received limited attention and was mainly performed on systems with a batch reactor. The proposed models concern mainly the transport phenomena and kinetics for the reaction in the liquid. Further research in modeling of flow reactors may help to improve systems meant for applications.

Although hydrolysis technology using complex metal hydrides (e.g., NaBH_4_ and AB) have found its way into UAVs and submarine applications, the majority of these applications are currently still driven by fossil fuels. These application may benefit from the installation of filters to obtain desired the hydrogen purity, efficient drainage of spent fuel, and operation conditions (humidity, vibrations, temperature *etc.*). All these improvements may affect the fuel cell operation and consequently, the success of the application. Additionally, further research is needed to create a more straightforward hydrolysis system preferably with on-board recycling of the spent fuel and more robustness towards operation conditions. Indeed, the advantages such as silent mode of operation and thermal signature can be quickly overshadowed by disadvantages such as limited space in a UAVs and the weight of both the energy storage system and the auxiliary system (which can easily account for roughly one third of the total weight of the aircraft). Therefore, any weight reduction of the hydrolysis technology that can be achieved will translate directly into an increase in flight duration.

## 5. Conclusions

After 20 years of intensive research on complex metal hydrides, a widespread commercial penetration of these hydrogen storage materials in technical applications is not in sight for the next years. The research attention over the last years has been given to fundamental understanding of crystal structures, reaction kinetics and thermodynamics of these materials as well as development of materials with high hydrogen content. The development of tank systems and demonstration projects with complex metal hydrides has not always been part of the past research activities because of the challenges that come along with engineering development.

Several technical barriers still exist for successful implementation of solid-state hydrogen storage systems based on thermolysis for stationary and portable applications and remain a significant challenge for transportation applications. Currently available storage tank systems typically require large volume, high weight, and have slow hydrogen charging/discharging rates and high system cost. Long-term research goals for the complex hydride materials should be to improve the gravimetric and volumetric capacities, to obtain sufficient kinetics within appropriate temperatures and pressure ranges and to lower the cost of the overall systems.

It is obvious that commercial success is not only a question of high hydrogen content of hydrogen storage materials. The overall performance of the whole system, consisting of hydrogen storage material, as well as storage tank design and integration of all the components into a technical system, plays a much more important role than one particular property. Taking this into account, engineering developments for the optimization of solid-state hydrogen storage tank systems must be intensified to overcome technical barriers and to construct safe and consumer-friendly system based on renewable energy carriers.
